# Polytherapy and Multimorbidity Pattern of Users of Anti-VEGF Drugs and Dexamethasone for the Treatment of Age-Related Macular Degeneration and other Vascular Retinopathies in Clinical Practice

**DOI:** 10.3390/ph16050646

**Published:** 2023-04-25

**Authors:** Ersilia Lucenteforte, Marco Finocchietti, Antonio Addis, Mauro Tettamanti, Monica Varano, Mariacristina Parravano, Gianni Virgili

**Affiliations:** 1 Department of Clinical and Experimental Medicine, University of Pisa, 56126 Pisa, Italy; ersilia.lucenteforte@unipi.it; 2 Department of Epidemiology, Lazio Regional Health Service, 00154 Rome, Italy; m.finocchietti@deplazio.it (M.F.); a.addis@deplazio.it (A.A.); 3 Department of Health Policy, Istituto di Ricerche Farmacologiche Mario Negri IRCCS, 20156 Milan, Italy; mauro.tettamanti@marionegri.it; 4 IRCCS Fondazione Bietti, 00198 Rome, Italy; monica.varano@fondazionebietti.it; 5 Department of NEUROFARBA, Eye Clinic, Careggi University Hospital, University of Florence, 50134 Florence, Italy; gianni.virgili@unifi.it

**Keywords:** retina, vascular diseases, antiangiogenic therapy, vascular endothelial growth factor, intravitreal injection, polypharmacy, multimorbidity

## Abstract

**Introduction:** Our aim was to describe the polytherapy and multimorbidity pattern of users of anti-VEGF and dexamethasone drugs for the treatment of these conditions, and to investigate their polytherapy and multimorbidity profiles, together with adherence and the burden of care. **Methods:** Descriptive, population-based, pharmacoepidemiology study on the users of anti-VEGF drugs, and secondarily intravitreal dexamethasone, for the treatment of age-related macular degeneration and other vascular retinopathies in clinical practice, using administrative databases of Lazio region, Italy. We used a cohort of 50,000 residents in Lazio in 2019 with same age as comparison. Polytherapy was assessed using databases of prescribed drugs intended for outpatient use. Multimorbidity was investigated with additional sources, such as hospital discharge records, outpatient care records, and disease-specific exemptions from co-payment. Each patient was followed for 1 to 3 years from the first intravitreal injection received. **Results:** 16,266 residents in Lazio who received the first IVI from 1 January 2011 to 31 December 2019, with at least 1 year of observation before index date, were included. The proportion of patients with at least one comorbidity was 54.0%. Patients used an average 8.6 (SD 5.3) concomitant drugs other than anti-VEGF used for injections. A large percentage of patients (39.0%) used 10 or more concomitant drugs, including antibacterials (62.9%), drugs for peptic ulcers (56.8%), anti-thrombotics (52.3%), NSAIDs (44.0%), and anti-dyslipidaemics (42.3%). The same proportions were found across patients of all ages, probably due to high prevalence of diabetes (34.3%), especially in younger age groups. When stratified by diabetes, a comparison of multimorbidity and polytherapy with a sample of 50,000 residents of the same age found that patients receiving IVIs used more drugs and had more comorbidities, particularly in non-diabetics. Lapses of care, whether short (absence of any type of contact for at least 60 days in the first year of follow-up and 90 in the second year) or long (90 days in the first and 180 days in the second year) were common: 66% and 51.7%, respectively. **Conclusions:** Patients receiving intravitreal drugs for retinal conditions have high multimorbidity and polytherapy rates. Their burden of care is aggravated by the large number of contacts with the eye care system for examinations and injections. Pursuing Minimally Disruptive Medicine to optimise patient care is a difficult goal for health systems, and more research on clinical pathways and their implementation is warranted.

## 1. Introduction

In the last decade, anti-angiogenic therapy, i.e., intravitreal injections of anti-vascular endothelial growth factor (anti-VEGF) agents, has been the standard of care for the treatment of neovascular eye diseases, particularly in age-related macular degeneration (AMD), diabetic retinopathy (DR) including diabetic macular edema (DME), and macular edema secondary to retinal vein occlusions (RVO). Among these, AMD is the leading cause of irreversible blindness in people 50 years of age or older in the developed world [1]. DR is the most prevalent retinal vascular disease and a severe ocular complication of diabetes mellitus. It is the leading cause of blindness in the working age population in developed countries [2]. RVO is the second most common retinal vascular disease after diabetic retinopathy [3] and a common cause of vision loss in older persons. RVO has a prevalence of 1 to 2% in persons older than 40 years of age and affects 16 million persons worldwide [4].

The most commonly used VEGF antagonists are ranibizumab, aflibercept, and off-label bevacizumab, but others have been recently approved, including ranibizumab biosimilars, brolucizumab, and faricimab [5]. Brolucizumab and faricimab have been approved by the EMA, but the former has only recently been introduced in the Italian market, and the latter is not yet reimbursed by the Italian regulatory body AIFA. Intravitreal dexamethasone is a different drug class (steroids) and is also approved in Italy for use in macular edema associated with DR or RVO in pseudophakic patients or as second line.

Patients treated for neovascular AMD and DME, as well as other retinal vascular diseases, are likely to suffer from multiple health conditions. Multimorbidity (the coexistence of multiple health conditions in an individual) and comorbidity (burden of illness co-existing with a particular disease of interest) are a growing global public health challenge as populations age and the prevalence of long-term conditions rises [6]. Multimorbidity and comorbidity are almost always accompanied by polytherapy. The term polytherapy refers to the use of various drugs, ranging typically from 5 to 10 [7], or the daily intake of five or more drugs [8], and it is often used to explicate the inappropriate use of multiple medications or simply more medications than needed by the patient.

Vision impairment is known to be highly prevalent in people with major multimorbidities [9]. Better knowledge of morbidity patterns may help in the development and implementation of interventions to avert the more serious consequences of having multiple chronic conditions, while minimizing the burden of care for these patients [10].

The current burden of multimorbidity and polytherapy in patients treated with anti-VEGF drugs for retinal vascular diseases is unknown. Thus, this study aimed at: (1) describing the polytherapy and multimorbidity pattern of users of anti-VEGF drugs for the treatment of age-related macular degeneration and other vascular retinopathies in clinical practice; (2) investigating the impact of polytherapy and multimorbidity on compliance, i.e., adherence to minimum requirements in terms of injections, examinations and follow-up duration.

## 2. Results

**Figure 1:**
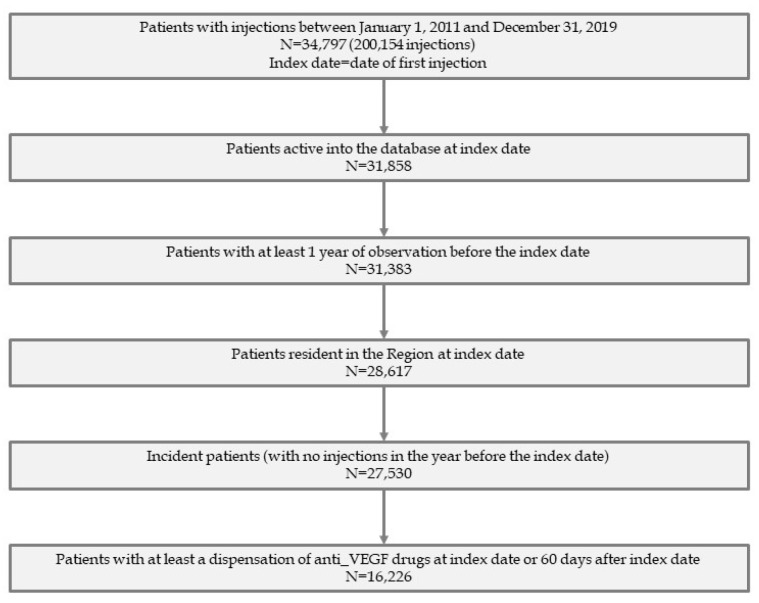
Flow chart of patient selection.

From 1 January 2011 to 31 December 2019, we identified 34,792 patients with intravitreal injections of anti-VEGFs. After considering patients active in the database at index date (31,858 patients), with at least 1 year of observation before index date (31,383), resident in Lazio at index date (28,617), and with no injection in the year before index date (27,530), we identified 16,266 patients with a first intravitreal injection of aflibercept, bevacizumab, dexamethasone, ranibizumab and pegaptanib between 2011 and 2019 in the Lazio Region (Figure 1).

A majority of patients used ranibizumab (53.8%, Table 1), followed by aflibercept (26.1%) and dexamethasone (15.4%). Very few patients used bevacizumab (3.6%) or pegaptanib (1.1%). Patients were mainly female (53%), with a mean age of 72.8 years (standard deviation, SD, 11.5), and most (48.0%) had completed middle school education. Users of dexamethasone appeared younger than others.

The pattern of use of intravitreal injections was consistent with previous findings of undertreatment [11,12]. Specifically, patients received 3.7 (SD 2.2), 1.3 (SD 2.0) and 1.0 (1.8) injections in the first, second and third year, respectively; higher numbers were observed for specialist examinations, i.e., 8.9 (SD 6.5), 3.7 (SD 4.9), and 2.8 (SD 4.4), respectively (Table 2). The number of injections during the three years was significantly higher in aflibercept users compared to other users, as was the number of specialist examinations compared to bevacizumab and dexamethasone users (*p*-values of all coefficients from adjusted linear regression models were lower than 0.001); moreover, aflibercept users had a significantly higher number of specialist examinations when compared to bevacizumab and dexamethasone users and a lower number when compared to ranibizumab and pegaptanib users in the first year, a higher number when compared to bevacizumab and dexamethasone users and a lower number when compared to pegaptanib users in the second year, and a higher number when compared to bevacizumab and dexamethasone users (*p*-values of all coefficients from adjusted linear regression models were lower than 0.001). The proportion of subjects with at least three doses in the first 90 days of follow-up was only 40.5% (Table 2). The proportion was significantly higher in aflibercept users compared to other users (*p*-values of all coefficients from adjusted logistic regression models were lower than 0.001). Patients were mainly non-compliant, as indicated by their lack of contact with healthcare providers for extended periods during the first and second years of follow-up (Table 2). Specifically, 66% had no contact for 60 days or more in the first year or 90 days or more in the second year, and 51.7% had no contact for 90 days or more in the first year or 180 days or more in the second year. Notably, the proportion of non-compliant patients was significantly higher among those using aflibercept as compared to other users when the first definition of non-compliance was adopted (no contact for 60 days or more in the first year or 90 days or more in the second year), while when we used the second definition (no contact for 90 days or more in the first year or 180 days or more in the second year), the proportion was significantly higher among aflibercept users only when compared to bevacizumab and dexamethasone users (*p*-values of all coefficients from adjusted logistic regression models were lower or equal to 0.001). Similar patterns were reported across all ages (Supplementary Materials) and both sexes (Supplementary Materials).

The proportion of patients with no history of comorbidity was 46.0% (Table 3). Psychiatric or mental health conditions were uncommon in the study population (all percentages were below or about 1%, Table 3), while cardio/cerebrovascular diseases were more common, in particular hypertension (30.6%). Diabetes was also frequently reported, consistent with the indications of these drugs (34.3%). Dexamethasone users seemed to report more frequently hypertension (35.0%) and diabetes (42.9%) compared with other users. Comorbidities were substantially reported by the same proportion of patients in all ages (Supplementary Materials), and no major differences emerged by gender (Supplementary Materials).

Patients used an average of 8.6 (SD 5.3) concomitant drugs other than anti-VEGF used for injections (Table 4). The number of concomitant drugs was significantly higher in dexamethasone and ranibizumab users as compared to aflibercept users (*p*-values of all coefficients from adjusted linear regression models were lower than 0.001). A large proportion of patients (39.0%) used 10 or more concomitant drugs (Table 4). This percentage seemed higher for dexamethasone (43.4%) and pegaptanib (43.6%) groups. Drugs more frequently reported were antibacterials (62.9%), drugs for peptic ulcers (56.8%), anti-thrombotics (52.3%), NSAIDs (44.0%), and anti-dyslipidaemics (42.3%). A similar pattern of use was reported at all ages (Supplementary Materials), and no major differences emerged by gender (Supplementary Materials).

Adherence and discontinuation were similar among 8025 patients (49.5%) with proxies of AMD, while polytherapy and comorbidity appeared to be less common (Supplementary Materials), similarly to non-diabetic patients of the entire cohort.

**Figure 2:**
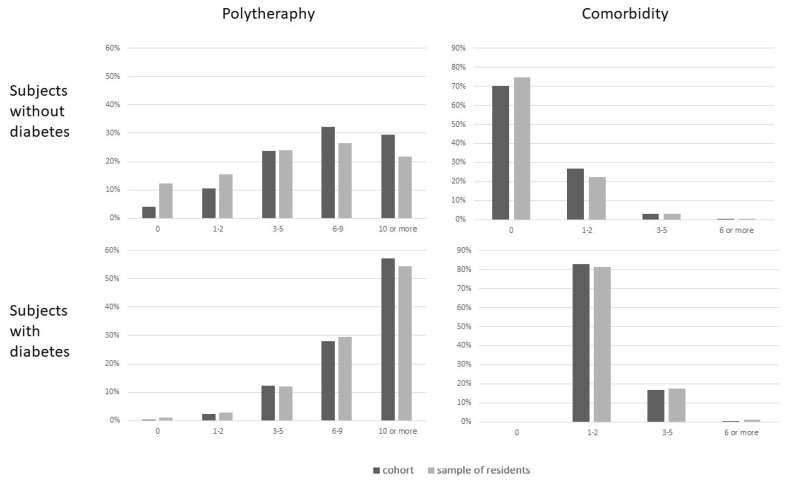
Comparison of our cohort with that of 50,000 residents in Lazio by polytherapy and comorbidity.

When we compared our cohort with a sample of 50,000 residents of the same age (Figure 2), we found that non-diabetic patients with a first intravitreal injection of anti-VEGF used more drugs and had more comorbidities compared with non-diabetic residents. Similar patterns was observed for patients with diabetes, although differences were reduced.

In the year before injection, 41.2% of patients had proxies of diabetes-related eye disease (Table 5). This percentage seemed to be higher (55.1%) for patients using dexamethasone as index drug compared with other index drugs, as expected given their approved indications. The percentage of patients with glaucoma was low (3.8% in the overall population), as was that with possible binocularity (9.9% overall). On average, these patients used two (SD 2.1) ophthalmic services before their first injection, mainly specialist encounters. Once more, similar patterns were reported across all ages (Supplementary Materials), and no major differences emerged by gender (Supplementary Materials).

## 3. Discussion

To the best of our knowledge, this is the first study investigating the multimorbidity and polytherapy profile of a large population of patients receiving intravitreal injections of anti-VEGF drugs and dexamethasone for retinal diseases. We expected these patients to represent a frail population. In fact, DME is a common indication for treatment and is typically associated with poor diabetic control and vascular morbidity. Moreover, AMD shares causal pathways with cardiovascular diseases and shows a bidirectional association with dementia and other neurologic diseases [13,14,15,16,17,18].

The finding that patients receiving anti-VEGF injection have high rates of multimorbidity and polytherapy was thus expected; nonetheless, we provided a confirmation and additional data. Particularly, more than half of the patients of all ages used six or more drugs, with a peak of about 75% between 75 and 80 years of age; at least half of these individuals used 10 or more drugs. The limited effect of age may be due to the fact that 40% to 45% of patients below age 70 were diabetic, a percentage that decreased to about 20% at age 85 or more. If age effects are disregarded, females were found to have a lower prevalence of diabetes than males (about 29% vs. 41%). The median number of drugs was six (IQR: 3–9) below age 65, eight (ICR: 5–12) at ages 65–75, and nine (IQR: 6—12/13) over 75 years of age.

The burden of care of these patients with multimorbidity is aggravated by having retinal diseases, since an average of 8.9, 3.4, and 2.4 ophthalmic contacts, including injections and/or examinations, were delivered in years 1, 2 and 3, respectively, with wide variation. Considering that only 3.7, 1.2, and 0.8 average injections were delivered in the first three years, respectively, and that only about 40% of patients received a loading dose of three injections, we confirmed that these patients were undertreated in Italy. The low average number of injections, far below the six to seven injections delivered in the UK for DMO [19] and AMD [20] in the first year, respectively, matches previous findings based on similar data in Tuscany [12].

Undertreatment was accompanied by low adherence. In fact, two-thirds of patients had short treatment lapses and about a half had long lapses, which were shown to be related to patients’ clinical status and outcome, as well as to their interaction with the health care system [21]. Of note, we found no association between low adherence and age or sex, hence suggesting no inequalities in this regard. We did not correlate adherence with socioeconomic status, since patient education was inconsistently collected in our databases, and no income data were available at the patient level. Finally, we did not focus on differences between drugs, although we acknowledge that they may have different indications, as reflected by slightly different diabetes prevalence, with a minimum of about 30% for aflibercept to 36% for bevacizumab for anti-VEGF drugs, and up to 42.9% for dexamethasone.

Our study has strengths and limitations. Among the former, it had region-wide population coverage of nearly 6 million individuals and collection of data using standardised criteria with almost a 10-year span. The main limitation is the lack of ocular diagnosis and laterality, which we managed using previously validated proxy indicators. This limitation may be more relevant if the focus is on ocular and vision outcomes, as compared to a broad investigation of multimorbidity, polytherapy and service use such as ours.

## 4. Materials and Methods

### 4.1. Study Design

This study was a descriptive, population-based, pharmacoepidemiology study on the users of anti-VEGF drugs for the treatment of age-related macular degeneration and other vascular retinopathies in clinical practice.

### 4.2. Setting

Italy has a tax-based, universal coverage National Health System organised in three levels: national; regional (20 regions); and local (on average 5 Local Health Units, LHUs, per region). Healthcare is managed for every inhabitant by the LHU, where they have their regular address. In the Lazio region up to 2020, there were 10 LHUs.

### 4.3. Data Sources

This study was based on the analysis of the Lazio databases, which collect pseudonymized patient-level information on the utilization of healthcare services dispensed to all subjects who are residents and registered with a general practitioner in the two regions, corresponding to a population of around 5.8 million people.

For each subject in the database, demographic information, such as age, sex and pertinent Local Health Authority, were linked to different registries in which different types of healthcare services reimbursed by the National Healthcare Service are recorded. These include:

▪ Inhabitant Registry (IR) with demographic information (birthyear, gender, citizenship), start and end dates of presence in the region;
▪ Hospital discharge records (HOSP): each hospital admission is described with dates of admission and discharge, and one main and five secondary diagnoses and 6 procedures coded using the International Classification of Diseases, Ninth Revision, Clinical Modification (ICD9CM);
▪ Outpatient care records (OUTPAT): it is a list of outpatient activities dispensed by the healthcare system free of charge or upon co-payment, among which specialist encounters (with no diagnostic code), laboratory or instrumental or bio-imaging diagnostic tests (without results) and procedures in outpatient setting are recorded with a specific Italian coding system; the facility where the activity takes place is recorded as well;
▪ Prescribed drugs intended for outpatient use. Prescription records include information on the dispensed drugs (e.g., active principle, ATC code) as well as the date of dispensation. Drugs are registered in two databases: one collects dispensing from hospital pharmacies (DDRUG), the other dispensing from community pharmacies (DRUGS);
▪ Disease-specific exemptions from co-payment to health care coding using ICD9CM (EXE).

### 4.4. Study Population

We considered all subjects with intravitreal injections recorded in OUTPAT of Lazio between 1 January 2011 and 31 December 2019 (see Supplementary Materials for patient selection scheme). We defined the first date of injection as index date and selected incident patients (patients without injections in the year before index date), patients active into the database, and patients with at least 365 days of observation before date of injection (look-back period).

Each record of intravitreal injection was associated with a specific drug if a dispensation of the drug in DDRUG occurred the same day of injection or within 60 days after injection. We considered the following drugs: bevacizumab, ranibizumab, pegaptanib, aflibercept, and dexamethasone (see Supplementary Materials for ATC codes). Brolucizumab, faricimab, and ranibizumab biosimilars were not present in this dataset, since they have been approved only recently. However, this should not have an impact on the profile of the population receiving intravitreal treatment.

### 4.5. Follow-Up

Each patient was followed for 1 to 3 years from index date (the first date of record in OUTPAT of intravitreal injection received).

### 4.6. Cohort of Residents

To compare our results with the general population, we used a random sample of 50,000 residents in Lazio in 2019 with the same age distribution as our cohort.

### 4.7. Study Variables

Each patient was characterized according to history of comorbidity and use of drugs in the 5 years before the index date. For comorbidity, we considered psychiatric or mental health conditions (i.e., anxiety, bipolar disease, delirium, dementia, depression, non-schizophrenic psychosis, schizophrenia), cardio/cerebrovascular diseases (i.e., arrhythmia, congestive heart failure, ischemic heart disease, stroke, hypertension, peripheral arterial disease, valve disorders, venous thrombosis embolism), and other conditions (i.e., COPD, diabetes, osteoporosis, Parkinson’s disease, epilepsy, hip fracture, pneumonia). We also considered number of comorbidities as continuous and categorized variables (0; 1–2; 3–5; 6 or more comorbidities). All subjects with ≥1 record in HOSP/EXE with a diagnosis/exemption of the disease of interest were considered as affected by the corresponding disease (see Supplementary Materials for codes). For concomitant drugs, we considered specific drugs (i.e., digoxin, NSAIDs, low dosage aspirin, antibacterial, anti-thrombotics, agents for peptic ulcers, organic nitrates, corticosteroids, antihypertensives, anti-dyslipidaemic agents) and a number of different drugs as continuous and categorized variables (0; 1–2; 3–5; 6–9; 10 or more different drugs). Number of different drugs was the number of dispensations with different ATC in DDRUG or DRUGS (see Supplementary Materials for codes).

We also characterized patients, in the year before the index date, according to the following variables: proxy of diabetes-related eye disease injections (i.e., history of diabetes, use of argon-laser, age less than 55 years); glaucoma; binocularity (3 injections in less than 55 days or 2 injections in less than 25 days); and use of ophthalmic service procedures and diagnosis.

Moreover, we considered three definitions of adherence in the follow-up period (number of injections; number of specialist examinations; number of subjects with at least 3 doses in the first 90 days of follow-up) and two definitions of discontinuation (at least 60 days without contact in the first year of follow-up and at least 90 days in the second; at least 90 days without contact in the first year of follow-up and at least 180 days in the second).

### 4.8. Other Variables

Each patient was characterized according to drug of first injection (aflibercept, bevacizumab, dexamethasone, pegaptanib, ranibizumab), age (as continuous and as 10-year categorical variables), and gender.

### 4.9. Statistical Analysis

We described subjects in terms of age, gender, citizenship, and education. We reported descriptive statistics for all of the above characteristics and of all variables of interest overall, according to drug of first injection, age, and gender. To investigate if type of drug influenced adherence, discontinuation, polytherapy, and comorbidities, we conducted linear and logistic regression analysis. All models were adjusted for gender, age and education.

As a sensitivity analysis of patients most likely to have AMD, we selected patients over 60 years of age, without diabetes and with no prescription of anticoagulant within 6 months of first injection, which we used as a proxy of RVO following Italian guidelines).

## 5. Conclusions

In conclusion, we have shown that patients receiving intravitreal anti-VEGF drugs and dexamethasone for retinal conditions have high multimorbidity and polytherapy rates. Their burden of care is aggravated by the large number of contacts with the eye care system for examinations and injections. Our data can be used by policy makers to plan clinical pathway guidance that aims to simultaneously increase the intensity of intravitreal treatments, while optimizing the burden of care due to multimorbidity and polytherapy. Pursuing Minimally Disruptive Medicine [10] is a difficult goal for health systems, and more research on clinical pathway optimization and implementation is warranted.

## Figures and Tables

**Table 1:** Sociodemographic characteristics at index date of patients, overall and by drug of first injection.

	Overall	Aflibercept	Bevacizumab	Dexamethasone	Ranibizumab	Pegaptanib
**TOTAL, N (%)**	16,226	4230 (26.1)	593 (3.6)	2500 (15.4)	8731 (53.8)	172 (1.1)
**Gender**						
Female, N (%)	8605 (53.0)	2327 (55.0)	311 (52.4)	1116 (44.6)	4766 (54.6)	85 (49.4)
**Age**						
mean (SD), years	72.8 (11.5)	74.5 (10.8)	72.6 (12.0)	69.7 (11.6)	72.9 (11.7)	74.6 (10.1)
groups, N (%)						
<55	1310 (8.1)	257 (6.0%)	54 (9.1)	267 (10.7)	724 (8.3)	8 (4.7)
55-<65	2194 (13.5)	459 (10.9)	74 (12.5)	476 (19.0)	1170 (13.4)	15 (8.7)
65-<75	4844 (29.9)	1183 (28.0)	175 (29.5)	856 (34.2)	2565 (29.4)	65 (37.8)
75-<85	5938 (36.6)	1742 (41.2)	225 (37.9)	747 (29.9)	3166 (36.3)	58 (33.7)
85+	1940 (12.0)	589 (13.9)	65 (11.0)	154 (6.2)	1106 (12.7)	26 (15.1)
**Education**						
None	507 (3.1)	153 (3.6)	19 (3.2)	64 (2.6)	268 (3.1)	3 (1.7)
Middle school	7782 (48.0)	1932 (45.7)	297 (50.1)	1184 (47.4)	4284 (49.1)	85 (49.4)
High school	3420 (21.1)	937 (22.2)	118 (19.9)	580 (23.2)	1743 (20.0)	42 (24.4)
College	1080 (6.7)	332 (7.8)	27 (4.6)	154 (6.2)	561 (6.4)	6 (3.5)
Unknown	3437 (21.2)	876 (20.7)	132 (22.3)	518 (20.7)	1875 (21.5)	36 (20.9)

**Table 2:** Adherence and discontinuation during follow-up.

	Overall	Aflibercept	Bevacizumab	Dexamethasone	Ranibizumab	Pegaptanib
**TOTAL, N (%)**	16,226	4230 (26.1)	593 (3.6)	2500 (15.4)	8731 (53.8)	172 (1.1)
**Adherence**						
Number of injections in the follow-up periods, mean (standard deviation)						
First year	3.7 (2.2)	4.5 (2.3)	3.5 (2.2)	2.0 (1.4)	3.8 (2.0)	3.6 (1.7)
Second year	1.3 (2.0)	1.6 (2.1)	1.3 (2.1)	0.9 (1.6)	1.3 (2.0)	1.0 (1.6)
Third year	1.0 (1.8)	1.3 (2.0)	0.8 (1.6)	0.6 (1.3)	1.0 (1.8)	0.6 (1.3)
Number of specialist examinations in the follow-up period, mean (standard deviation)						
First year	8.9 (6.5)	9.1 (6.3)	6.5 (4.7)	6.7 (4.7)	9.5 (6.9)	12.7 (8.0)
Second year	3.7 (4.9)	3.6 (4.7)	2.7 (3.7)	3.2 (4.2)	3.8 (5.3)	5.3 (6.8)
Third year	2.8 (4.4)	2.9 (4.4)	1.8 (2.9)	2.2 (3.4)	2.9 (4.6)	3.4 (4.8)
Number of subjects with at least 3 doses in the first 90 days of follow-up	6569 (40.5)	2229 (52.7)	172 (29.0)	35 (1.4)	4097 (46.9)	36 (20.9)
**Discontinuation, N (%)**						
At least 60 days without contact in the first year of follow-up and at least 90 days in the second	10,706 (66.0)	3219 (76.1)	349 (58.9)	1424 (57.0)	5601 (64.2)	113 (65.7)
At least 90 days without contact in the first year of follow-up and at least 180 days in the second	8384 (51.7)	2225 (52.6)	262 (44.2)	1393 (55.7)	4421 (50.6)	83 (48.3)

**Table 3:** History of comorbidities, overall and by drug of first injection.

	Overall	Aflibercept	Bevacizumab	Dexamethasone	Ranibizumab	Pegaptanib
**TOTAL, N (%)**	16,226	4230 (26.1)	593 (3.6)	2500 (15.4)	8731 (53.8)	172 (1.1)
**Charlson Comorbidity Index**						
Mean (standard deviation)	0.5 (1.2)	0.4 (1.1)	0.4 (1.1)	0.6 (1.4)	0.4 (1.2)	0.4 (1.0)
**Number of comorbidities (one of those present in Table**)						
Mean (standard deviation)	0.9 (1.1)	0.8 (1.0)	0.8 (1.0)	1.0 (1.1)	0.9 (1.0)	0.9 (1.1)
Median (interquartile range)	1.0 (0.0–1.0)	1.0 (0.0–1.0)	1.0 (0.0–1.0)	1.0 (0.0–2.0)	1.0 (0.0–1.0)	0.0 (0.0–2.0)
Group, N (%)						
0	7471 (46.0)	2049 (48.4)	284 (47.9)	974 (39.0)	4076 (46.7)	88 (51.2)
1–2	7468 (46.0)	1866 (44.1)	270 (45.5)	1271 (50.8)	3992 (45.7)	69 (40.1)
3–5	1253 (7.7)	307 (7.3)	39 (6.6)	249 (10.0)	643 (7.4)	15 (8.7)
6+	34 (0.2)	8 (0.2)	-	6 (0.2)	20 (0.2)	-
**Psychiatric or mental health conditions, N (%)**						
Anxiety	132 (0.8)	25(0.6)	4 (0.7)	20 (0.8)	83 (1.0)	-
Bipolar disease	27 (0.2)	8 (0.2)	1 (0.2)	3 (0.1)	15 (0.2)	-
Delirium	15 (0.1)	1 (0.0)	-	4 (0.2)	10 (0.1)	-
Dementia	28 (0.2)	7 (0.2)	1 (0.2)	6 (0.2)	13 (0.1)	1 (0.6)
Depression	46 (0.3)	7 (0.2)	-	9 (0.4)	30 (0.3)	-
Non-schizophrenic psychosis	29 (0.2)	7 (0.2)	-	2 (0.1)	20 (0.2)	-
Schizophrenia	17 (0.1)	2 (0.0)	-	3 (0.1)	12 (0.1)	-
**Cardio/cerebrovascular diseases, N (%)**						
Arrhythmia	251 (1.5)	81 (1.9)	2 (0.3)	44 (1.8)	123 (1.4)	1 (0.6)
Congestive heart failure	323 (2.0)	80 (1.9)	13 (2.2)	49 (2.0)	178 (2.0)	3 (1.7)
Ischemic heart disease	1166 (7.2)	302 (7.1)	29 (4.9)	204 (8.2)	608 (7.0)	23 (13.4)
Stroke	224 (1.4)	60 (1.4)	7 (1.2)	46 (1.8)	108 (1.2)	3 (1.7)
Hypertension	4961 (30.6)	1262 (29.8)	185 (31.2)	875 (35.0)	2583 (29.6)	56 (32.6)
Peripheral arterial disease	28 (0.2)	11 (0.3)	-	7 (0.3)	10 (0.1)	-
Valve disorders	224 (1.4)	54 (1.3)	5 (0.8)	36 (1.4)	127 (1.5)	2 (1.2)
Venous thrombosis embolism	75 (0.5)	12 (0.3)	1 (0.2)	28 (1.1)	33 (0.4)	1 (0.6)
**Other conditions, N (%)**						
Diabetes	5561 (34.3)	1287 (30.4)	215 (36.3)	1072 (42.9)	2941 (33.7)	46 (26.7)
COPD	373 (2.3)	102 (2.4)	10 (1.7)	52 (2.1)	204 (2.3)	5 (2.9)
Osteoporosis	106 (0.7)	34 (0.8)	5 (0.8)	10 (0.4)	56 (0.6)	1 (0.6)
Parkinson’s disease	38 (0.2)	7 (0.2)	-	11 (0.4)	19 (0.2)	1 (0.6)
Epilepsy	67 (0.4)	23 (0.5)	2 (0.3)	14 (0.6)	26 (0.3)	2 (1.2)
Hip fracture	35 (0.2)	10 (0.2)	1 (0.2)	4 (0.2)	20 (0.2)	-
Pneumonia	316 (1.9)	81 (1.9)	6 (1.0)	51 (2.0)	176 (2.0)	2 (1.2)

**Table 4:** History of concomitant drugs, overall and by drug of first injection.

	Overall	Aflibercept	Bevacizumab	Dexamethasone	Ranibizumab	Pegaptanib
**TOTAL, N (%)**	16,226	4230 (26.1)	593 (3.6)	2500 (15.4)	8731 (53.8)	172 (1.1)
**Number of concomitant drugs**						
Mean (standard deviation)	8.6 (5.3)	8.4 (5.0)	8.5 (5.1)	9.3 (5.4)	8.6 (5.3)	9.2 (5.8)
Median (interquartile range	8.0 (5.0–12.0)	8.0 (5.0–11.0)	8.0 (5.0–11.0)	9.0 (5.0–12.0)	8.0 (5.0–12.0)	8.5 (5.0–12.0)
Group, N (%)						
0	447 (2.8)	122 (2.9)	20 (3.4)	39 (1.6)	260 (3.0)	6 (3.5)
1–2	1249 (7.7)	336 (7.9)	49 (8.3)	148 (5.9)	711 (8.1)	5 (2.9)
3–5	3202 (19.7)	835 (19.7)	102 (17.2)	484 (19.4)	1746 (20.0)	35 (20.3)
6–9	4992 (30.8)	1369 (32.4)	205 (34.6)	745 (29.8)	2622 (30.0)	51 (29.7)
10+	6336 (39.0)	1568 (37.1)	217 (36.6)	1084 (43.4)	3392 (38.9)	75 (43.6)
**Type of concomitant drug (ATC code), N (%)**						
Digoxin (C01AA05)	300 (1.8)	63 (1.5)	14 (2.4)	32 (1.3)	187 (2.1)	4 (2.3)
NSAIDs (M01)	7137 (44.0)	1791 (42.3)	268 (45.2)	1049 (42.0)	3942 (45.1)	87 (50.6)
Low-dose aspirin (B01AC06; B01AC30	5760 (35.5)	1467 (34.7)	205 (34.6)	1069 (42.8)	2951 (33.8)	68 (39.5)
Antibacterial (J01)	10,207 (62.9)	2573 (60.8)	387 (65.3)	1584 (63.4)	5536 (63.4)	127 (73.8)
Anti-thrombotic (B01)	8486 (52.3)	2170 (51.3)	294 (49.6)	1628 (65.1)	4296 (49.2)	98 (57.0)
Drugs for peptic ulcers (A02)	9209 (56.8)	2349 (55.5)	323 (54.5)	1451 (58.0)	4987 (57.1)	99 (57.6)
Organic nitrates (C01DA)	815 (5.0)	176 (4.2)	40 (6.7)	106 (4.2)	474 (5.4)	19 (11.0)
Corticosteroids (H02)	2846 (17.5)	749 (17.7)	84 (14.2)	562 (22.5)	1425 (16.3)	26 (15.1)
Antihypertensives (C02)	1059 (6.5)	244 (5.8)	41 (6.9)	192 (7.7)	572 (6.6)	10 (5.8)
Anti-dyslipidaemic agents (C10)	6863 (42.3)	1779 (42.1)	239 (40.3)	1100 (44.0)	3660 (41.9)	85 (49.4)

**Table 5:** History of proxy of diabetes-related eye disease, glaucoma, use of ophthalmic services and binocularity, overall and by drug of first injection.

	Overall	Aflibercept	Bevacizumab	Dexamethasone	Ranibizumab	Pegaptanib
**TOTAL, N (%)**	16,226	4230 (26.1)	593 (3.6)	2500 (15.4)	8731 (53.8)	172 (1.1)
**History of proxy of diabetes-related eye disease, N (%)**						
Diabetes	5545 (34.2)	1283 (30.3)	214 (36.1)	1071 (42.8)	2931 (33.6)	46 (26.7)
Argon-laser retina (and laser photocoagulation)	1205 (7.4)	164 (3.9)	61 (10.3)	345 (13.8)	622 (7.1)	13 (7.6)
Younger than 55 at first injection	1310 (8.1)	257 (6.1)	54 (9.1)	267 (10.7)	724 (8.3)	8 (4.7)
Any proxy among the previous	6679 (41.2)	1478 (34.9)	267 (45.0)	1378 (55.1)	3501 (40.1)	55 (32.0)
**History of glaucoma, N (%)**	617 (3.8)	146 (3.5)	26 (4.4)	79 (3.2)	360 (4.1)	6 (3.5)
**History of use of ophthalmic services, Mean (SD)**						
Specialist encounter	2.0 (2.1)	1.6 (1.8)	1.5 (1.8)	2.3 (2.3)	2.1 (2.1)	3.2 (2.8)
OCT	0.0 (0.0)	0.0 (0.0)	0.0 (0.0)	0.0 (0.0)	0.0 (0.0)	0.0 (0.0)
Fluorescence imaging	0.7 (0.9)	0.5 (0.8)	0.6 (0.7)	0.6 (0.8)	0.7 (0.9)	1.0 (1.0)
Fluorescence imaging with indocyanine	0.0 (0.0)	0.0 (0.0)	0.0 (0.0)	0.0 (0.0)	0.0 (0.0)	0.0 (0.0)
Fundus photography	0.0 (0.2)	0.0 (0.2)	0.0 (0.1)	0.0 (0.2)	0.0 (0.1)	0.0 (0.2)
**Possible binocularity during the first year of follow-up period, N (%)**	1612 (9.9)	531 (12.6)	76 (12.8)	167 (6.7)	828 (9.5)	10 (5.8)

## Supplementary Materials

The following supporting information can be downloaded at: https://www.mdpi.com/article/10.3390/ph16050646/s1, Figure S1. Patient selection scheme; Table S1. Codes for intravitreal injection (ATC), comorbidity (ICD9M and EXE), concomitant drugs (ATC), and Injections, procedures and diagnostics; Table S2. Comorbidities by age; Table S3. Comorbidities by gender; Table S4. Polytherapy by age; Table S5. Polytherapy by gender; Table S6. Adherence and discontinuation during follow-up for patients with proxy od AMD; Table S7. History of comorbidities for patients with proxy od AMD. Table S8. History of concomitant drugs for patients with proxy od AMD. Table S9. History of proxy of diabetes-related eye disease, glaucoma, use of ophthalmic services and binocularity by age. Table S10. History of proxy of diabetes-related eye disease, glaucoma, use of ophthalmic services and binocularity by gender.

## References

[1] Resnikoff S., Pascolini D., Etya’Ale D., Kocur I., Pararajasegaram R., Pokharel G.P., Mariotti S.P. (2004). Global data on visual impairment in the year 2002. Bull. World Health Organ..

[2] Frank R.N. (2004). Diabetic Retinopathy. N. Engl. J. Med..

[3] Campochiaro P.A., Heier J.S., Feiner L., Gray S., Saroj N., Rundle A.C., Murahashi W.Y., Rubio R.G. (2010). Ranibizumab for Macular Edema following Branch Retinal Vein Occlusion: Six-Month Primary End Point Results of a Phase III Study. Ophthalmology.

[4] Rogers S., McIntosh R.L., Cheung N., Lim L., Wang J.J., Mitchell P., Kowalski J.W., Nguyen H., Wong T.Y. (2010). The Prevalence of Retinal Vein Occlusion: Pooled Data from Population Studies from the United States, Europe, Asia, and Australia. Ophthalmology.

[5] Servillo A., Zucchiatti I., Sacconi R., Parravano M., Querques L., La Rubia P., Prascina F., Bandello F., Querques G. (2022). The state-of-the-art pharmacotherapeutic management of neovascular age-related macular degeneration. Expert Opin. Pharmacother..

[6] Leal J.R., Laupland K.B. (2010). Validity of ascertainment of co-morbid illness using administrative databases: A systematic review. Clin. Microbiol. Infect..

[7] Ferner R.E., Aronson J.K. (2006). Communicating information about drug safety. Br. Med. J..

[8] Veehof L.J.G., Stewart R.E., Haaijer-Ruskamp F.M., Meyboom-de Jong B. (2000). The development of polypharmacy. A longitudinal study. Fam. Pract..

[9] Zheng D.D., Christ S.L., Lam B.L., Feaster D.J., McCollister K., Lee D.J. (2020). Patterns of Chronic Conditions and Their Association With Visual Impairment and Health Care Use. JAMA Ophthalmol..

[10] Boehmer K.R., Abu Dabrh A.M., Gionfriddo M.R., Erwin P., Montori V.M. (2018). Does the chronic care model meet the emerging needs of people living with multimorbidity? A systematic review and thematic synthesis. PLoS ONE.

[11] Scondotto G., Sultana J., Ientile V., Ingrasciotta Y., Fontana A., Copetti M., Mirabelli E., Trombetta C.J., Rapisarda C., Reibaldi M. (2020). How Have Intravitreal Anti-VEGF and Dexamethasone Implant Been Used in Italy? A Multiregional, Population-Based Study in the Years 2010–2016. BioMed Res. Int..

[12] Virgili G., Tosi G.M., Figus M., Rizzo S., Murro V., Mucciolo D.P., Roberto G., Gini R. (2019). Use of anti-vascular endothelial growth factor drugs for eye disease in Tuscany: Development and test of indicators of treatment intensity. Eur. J. Ophthalmol..

[13] Velilla S., Garcia-Medina J.J., García-Layana A., Dolz-Marco R., Pons-Vázquez S., Pinazo-Duran M.D., Gómez-Ulla F., Arevalo J.F., Díaz-Llopis M., Gallego-Pinazo R. (2013). Smoking and Age-Related Macular Degeneration: Review and Update. J. Ophthalmol..

[14] Adams M.K.M., Chong E.W., Williamson E., Aung K.Z., Makeyeva G.A., Giles G., English D., Hopper J., Guymer R., Baird P. (2012). 20/20—Alcohol and Age-related Macular Degeneration: The Melbourne Collaborative Cohort Study. Am. J. Epidemiol..

[15] Choi D., Choi S., Park S.M. (2018). Effect of smoking cessation on the risk of dementia: A longitudinal study. Ann. Clin. Transl. Neurol..

[16] Ngandu T., Lehtisalo J., Solomon A., Levälahti E., Ahtiluoto S., Antikainen R., Bäckman L., Hänninen T., Jula A., Laatikainen T. (2015). A 2 year multidomain intervention of diet, exercise, cognitive training, and vascular risk monitoring versus control to prevent cognitive decline in at-risk elderly people (FINGER): A randomised controlled trial. Lancet.

[17] Bettiol S.S., Rose T.C., Hughes C.J., Smith L.A. (2015). Alcohol Consumption and Parkinson’s Disease Risk: A Review of Recent Findings. J. Park. Dis..

[18] Carrière I., Delcourt C., Daien V., Pérès K., Féart C., Berr C., Ancelin M.L., Ritchie K. (2013). A prospective study of the bi-directional association between vision loss and depression in the elderly. J. Affect. Disord..

[19] Talks S.J., Stratton I., Peto T., Lotery A., Chakravarthy U., Eleftheriadis H., Izadi S., Dhingra N., Scanlon P., Talks J. (2021). Aflibercept in clinical practice; visual acuity, injection numbers and adherence to treatment, for diabetic macular oedema in 21 UK hospitals over 3 years. Eye.

[20] Mehta H., Kim L.N., Mathis T., Zalmay P., Ghanchi F., Amoaku W.M., Kodjikian L. (2020). Trends in Real-World Neovascular AMD Treatment Outcomes in the UK. Clin. Ophthalmol..

[21] Ehlken C., Ziemssen F., Eter N., Lanzl I., Kaymak H., Lommatzsch A., Schuster A.K. (2020). Systematic review: Non-adherence and non-persistence in intravitreal treatment. Graefe’s Arch. Clin. Exp. Ophthalmol..

