# Supplementary material for: Polytherapy and Multimorbidity Pattern of Users of Anti-VEGF Drugs and Dexamethasone for the Treatment of Age-Related Macular Degeneration and other Vascular Retinopathies in Clinical Practice
Source: Pharmaceuticals (Basel). 2023 Apr 25;16(5):646. doi: 10.3390/ph16050646 (PMC10222997; doi:10.3390/ph16050646)
Supplement: Supplementary file 1 [file pharmaceuticals-16-00646-s001.zip › pharmaceuticals-2298833-supplementary.pdf]

Supplementary Material: Polytherapy and multimorbidity pattern of users of anti-VEGF drugs and dexamethasone for the treatment of age-related macular degeneration and other vascular retinopathies in clinical practice

Ersilia Lucenteforte[1], Marco Finocchietti[2], Antonio Addis[2], Mauro Tettamanti[3], Monica Varano[4], Mariacristina Parravano[4,*], and Gianni Virgili[5].

[1]Department of Clinical and Experimental Medicine, University of Pisa, Pisa, Italy

[2]Department of Epidemiology, Lazio Regional Health Service, Rome, Italy

[3] Istituto di Ricerche Farmacologiche Mario Negri, Department of Health Policy, Milan, Italy

[4] IRCCS Fondazione Bietti, Rome, Italy

[5] Department of NEUROFARBA, Eye Clinic, Careggi University Hospital, University of Florence, Florence, Italy

Figure S1. Patient selection scheme 2

Table S1. Codes for intravitreal injection (ATC), comorbidity (ICD9M and EXE), concomitant drugs (ATC), and Injections, procedures and diagnostics....................2

Table S2. Comorbidities by age....................4

Table S3. Comorbidities by gender....................5

Table S4. Polytherapy by age 6

Table S5. Polytherapy by gender 7

Table S6. Adherence and discontinuation during follow-up for patients with proxy od AMD. 8

Table S7. History of comorbidities for patients with proxy od AMD.....................9

Table S8. History of concomitant drugs for patients with proxy od AMD. 10

Table S9. History of proxy of diabetes-related eye disease, glaucoma, use of ophthalmic services and binocularity by age....................11

Table S10. History of proxy of diabetes-related eye disease, glaucoma, use of ophthalmic services and binocularity by gender......12

Figure S1. Patient selection scheme

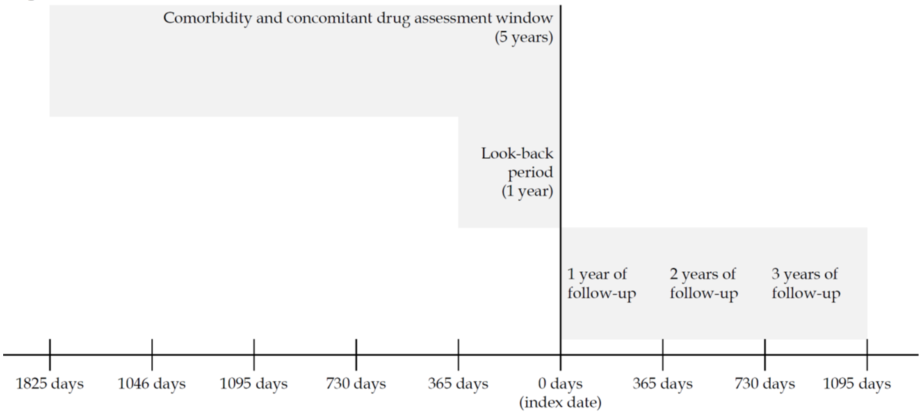

Table S1. Codes for intravitreal injection (ATC), comorbidity (ICD9M and EXE), concomitant drugs (ATC), and Injections, procedures and diagnostics

**Intravitreal injection (from DDRUG)**	**Code**
bevacizumab	L01XC07<br>0G00126001 (galenic preparation)
ranibizumab	S01LA04
pegaptanib	S01LA03
aflibercept	S01LA05, L01XX44
dexamethasone	A01AC02<br>C05AA09<br>D07AB19<br>D07XB05<br>D10AA03<br>H02AB02<br>H02AB02<br>R01AD03<br>S01BA01<br>S01CB01<br>S02BA06<br>S03BA01
**Comorbidity (from HOSP/EXE)**	
Psychiatric or mental health conditions	
Anxiety	300
Bipolar disease	296.00-296.06<br>296.4-296.8<br>EXE: 044.296.4-044.296.7
Delirium	293<br>297.1<br>780.09
Dementia	290.0-290.43<br>294.8<br>EXE: 011.290.0-011.290.4
Depression	296.2-296.4<br>311<br>EXE: 044.296.2-044.296.3
Non-schizophrenic psychosis	298.x<br>EXE: 044.297-0.44.299
Schizophrenia	295.x<br>EXE: 044.295.x
Cardio/cerebrovascular diseases	
Arrhythmia	427.60<br>427.9<br>EXE: 0A02.427
Congestive heart failure	428.0<br>EXE: 021.428
Ischaemic heart disease	414.8-414.9<br>EXE: 0A02.414, 002.414

Stroke	430<br>431<br>432.x<br>434.x<br>EXE: 0B02.433-0B02.437
Hypertension	401.0-405.9<br>EXE: 0A31, 031.x, 0031.x
Peripheral arterial disease	444.22<br>EXE: 0C02.444
Valve disorders	394-397<br>424.x<br>EXE: 0A02.394-0A02.397
Venous thrombosis embolism	452.x-453.x<br>EXE: 0C02.452-0C02.453
Other conditions	
Diabetes of use of antidiabetics (ATC: A10*)	250<br>EXE: 013.250
COPD	491.2<br>496<br>EXE: 057
Osteoporosis	733.x
Parkinson's disease	332.x<br>EXE: 038.333.0-038.333.1
Epilepsy	345.x<br>EXE: RF0060, 017.345
Hip fracture	808.x
Pneumonia	480-486
Glaucoma or use of antiglaucomatous medication (ATC: S01*)	
**Concomitant drugs (from DDRUG)**	
Digoxin	C01AA05
NSAIDs	M01
Low Dosage Aspirin	B01AC06<br>B01AC30
Antibacterial	J01
Anti-thrombotic	B01
Agents for peptic ulcers	A02
Organic nitrates	C01DA
Corticosteroids	H02
Antihypertensives	C02
Anti-dyslipidaemic agents	C10
**Injections, procedures and diagnostics (from OUTPAT)**	
Argon-laser	14.34<br>14.24.1
Specialist encounter	Visit: 95.02<br>or other vistis: 95.01<br>or control visits: 89.01
OCT	95.17
Fluorescence imaging	95.12
Fluorescence imaging with indocyanine	95.12.1
Laser photocoagulation	
binocularity	(2 injections within 25 days considering the old "catalogo prestanzioni ambulatoriali"
injections	14.79.1
Fundus photographs	95.11.1

Table S2. Comorbidities by age

	64-		65-69		70-74		75-79		80-84		85+	
**TOTAL**	3504		2026		2818		3174		2764		1940	
**Charlson Comorbidity Index**												
mean (SD)	0.4	1.2	0.5	1.3	0.5	1.2	0.4	1.2	0.4	1.2	0.4	1.0
**Number of comorbidities (one of those present in Table)**												
0	1688	48.2%	793	39.1%	1253	44.5%	1479	46.6%	1299	47.0%	959	49.4%
1-2	1594	45.5%	1047	51.7%	1332	47.3%	1452	45.7%	1239	44.8%	804	41.4%
3-5	215	6.1%	182	9.0%	226	8.0%	236	7.4%	220	8.0%	174	9.0%
6+	7	0.2%	4	0.2%	7	0.2%	7	0.2%	6	0.2%	3	0.2%
**Psychiatric or mental health conditions, N (%)**												
Anxiety	39	1.1%	13	0.6%	25	0.9%	27	0.9%	16	0.6%	12	0.6%
Bipolar disease	9	0.3%	4	0.2%	2	0.1%	9	0.3%	3	0.1%	0	0.0%
Delirium	3	0.1%	4	0.2%	2	0.1%	2	0.1%	3	0.1%	1	0.1%
Dementia	0	0.0%	0	0.0%	3	0.1%	11	0.3%	6	0.2%	8	0.4%
Depression	15	0.4%	6	0.3%	7	0.2%	11	0.3%	5	0.2%	2	0.1%
Non-schizophrenic psychosis	10	0.3%	5	0.2%	3	0.1%	5	0.2%	3	0.1%	3	0.2%
Schizophrenia	13	0.4%	0	0.0%	2	0.1%	1	0.0%	1	0.0%	0	0.0%
**Cardio/cerebrovascular diseases, N(%)**												
Arrhythmia	28	0.8%	27	1.3%	29	1.0%	65	2.0%	58	2.1%	44	2.3%
Congestive heart failure	51	1.5%	33	1.6%	52	1.8%	50	1.6%	62	2.2%	75	3.9%
Ischemic heart disease	169	4.8%	164	8.1%	231	8.2%	236	7.4%	207	7.5%	159	8.2%
Stroke	38	1.1%	36	1.8%	26	0.9%	43	1.4%	51	1.8%	30	1.5%
Hypertension	798	22.8%	676	33.4%	900	31.9%	1033	32.5%	924	33.4%	630	32.5%
Peripheral arterial disease	6	0.2%	7	0.3%	6	0.2%	5	0.2%	2	0.1%	2	0.1%
Valve disorders	24	0.7%	25	1.2%	37	1.3%	42	1.3%	53	1.9%	43	2.2%
Venous thrombosis embolism	40	1.1%	5	0.2%	7	0.2%	6	0.2%	11	0.4%	6	0.3%
**Other conditions, N(%)**												
Diabetes	1432	40.9%	919	45.4%	1069	37.9%	970	30.6%	763	27.6%	408	21.0%
COPD	26	0.7%	36	1.8%	54	1.9%	74	2.3%	98	3.5%	85	4.4%
Osteoporosis	10	0.3%	7	0.3%	19	0.7%	18	0.6%	21	0.8%	31	1.6%
Parkinson’s disease	3	0.1%	5	0.2%	5	0.2%	6	0.2%	6	0.2%	13	0.7%
Epilepsy	19	0.5%	9	0.4%	11	0.4%	11	0.3%	11	0.4%	6	0.3%
Hip fracture	2	0.1%	3	0.1%	4	0.1%	5	0.2%	15	0.5%	6	0.3%
Pneumonia	55	1.6%	26	1.3%	49	1.7%	67	2.1%	63	2.3%	56	2.9%

Table S3. Comorbidities by gender

	Male		Female	
**TOTAL**	7621		8605	
**Charlson Comorbidity Index**				
mean (SD)	0.6	(1.3)	0.4	(1.0)
**Number of comorbidities (one of those present in Table)**				
0	3020	39.6%	4451	51.7%
1-2	3797	49.8%	3671	42.7%
3-5	777	10.2%	476	5.5%
6+	27	0.4%	7	0.1%
**Psychiatric or mental health conditions, N (%)**				
Anxiety	45	0.6%	87	1.0%
Bipolar disease	9	0.1%	18	0.2%
Delirium	5	0.1%	10	0.1%
Dementia	12	0.2%	16	0.2%
Depression	14	0.2%	32	0.4%
Non-schizophrenic psychosis	11	0.1%	18	0.2%
Schizophrenia	6	0.1%	11	0.1%
**Cardio/cerebrovascular diseases, N(%)**				
Arrhythmia	156	2.0%	95	1.1%
Congestive heart failure	176	2.3%	147	1.7%
Ischemic heart disease	829	10.9%	337	3.9%
Stroke	131	1.7%	93	1.1%
Hypertension	2496	32.8%	2465	28.6%
Peripheral arterial disease	18	0.2%	10	0.1%
Valve disorders	118	1.5%	106	1.2%
Venous thrombosis embolism	41	0.5%	34	0.4%
**Other conditions, N(%)**				
Diabetes	3100	40.7%	2461	28.6%
COPD	223	2.9%	150	1.7%
Osteoporosis	27	0.4%	79	0.9%
Parkinson’s disease	23	0.3%	15	0.2%
Epilepsy	30	0.4%	37	0.4%
Hip fracture	11	0.1%	24	0.3%
Pneumonia	197	2.6%	119	1.4%

Table S4. Polytherapy by age

	64-		65-69		70-74		75-79		80-84		85+	
**TOTAL**	3504		2026		2818		3174		2764		1940	
**Concomitant drugs**												
Mean ± SD	6.5 ± 5.0		8.6 ± 5.2		9.0 ± 5.2		9.3 ± 5.2		9.7 ± 5.2		9.5 ± 5.2	
Median (IQR)	6.0 (3.0 - 9.0)		8.0 (5.0 - 12.0)		8.0 (5.0 - 12.0)		9.0 (6.0 - 12.0)		9.0 (6.0 - 13.0)		9.0 (6.0 - 12.0)	
Min - Max	0.0 - 29.0		0.0 - 39.0		0.0 - 35.0		0.0 - 37.0		0.0 - 46.0		0.0 - 52.0	
**Number of different drugs, N (%)**												
0	268	7.6%	40	2.0%	52	1.8%	41	1.3%	24	0.9%	22	1.1%
1-2	532	15.2%	171	8.4%	181	6.4%	169	5.3%	116	4.2%	80	4.1%
3-5	961	27.4%	397	19.6%	535	19.0%	573	18.1%	430	15.6%	306	15.8%
6-9	887	25.3%	625	30.8%	861	30.6%	1051	33.1%	916	33.1%	652	33.6%
10+	856	24.4%	793	39.1%	1189	42.2%	1340	42.2%	1278	46.2%	880	45.4%
**Type of drug, N (%)**												
Digoxin	15	0.4%	12	0.6%	27	1.0%	58	1.8%	89	3.2%	99	5.1%
NSAIDs	1216	34.7%	915	45.2%	1336	47.4%	1489	46.9%	1331	48.2%	850	43.8%
Low dosage aspirin	761	21.7%	716	35.3%	1096	38.9%	1239	39.0%	1154	41.8%	794	40.9%
Antibacterial	2042	58.3%	1302	64.3%	1829	64.9%	2027	63.9%	1781	64.4%	1226	63.2%
Anti-thrombotic	1184	33.8%	971	47.9%	1510	53.6%	1832	57.7%	1735	62.8%	1254	64.6%
Drugs for peptic ulcers	1366	39.0%	1112	54.9%	1672	59.3%	1939	61.1%	1831	66.2%	1289	66.4%
Organic nitrates	64	1.8%	80	3.9%	124	4.4%	147	4.6%	187	6.8%	213	11.0%
Corticosteroids	563	16.1%	356	17.6%	498	17.7%	600	18.9%	523	18.9%	306	15.8%
Antihypertensives	149	4.3%	138	6.8%	212	7.5%	237	7.5%	196	7.1%	127	6.5%
Anti-dyslipidaemic agents	1035	29.5%	938	46.3%	1360	48.3%	1491	47.0%	1284	46.5%	755	38.9%

Table S5. Polytherapy by gender

	Male		Female	
**TOTAL**	7621		8605	
**Concomitant drugs**				
Mean ± SD	8.5 ± 5.3		8.8 ± 5.2	
Median (IQR)	8.0 (5.0 - 12.0)		8.0 (5.0 - 12.0)	
Min - Max	0.0 - 46.0		0.0 - 52,0	
**Number of different drugs, N (%)**				
0	228	3.0%	219	2.5%
1-2	614	8.1%	635	7.4%
3-5	1565	20.5%	1637	19.0%
6-9	2322	30.5%	2670	31.0%
10+	2892	37.9%	3444	40.0%
**Type of drug, N (%)**				
Digoxin	121	1.6%	179	2.1%
NSAIDs	2942	38.6%	4195	48.8%
Low dosage aspirin	2961	38.9%	2799	32.5%
Antibacterial	4669	61.3%	5538	64.4%
Anti-thrombotic	4272	56.1%	4214	49.0%
Drugs for peptic ulcers	4138	54.3%	5071	58.9%
Organic nitrates	440	5.8%	375	4.4%
Corticosteroids	1213	15.9%	1633	19.0%
Antihypertensives	583	7.6%	476	5.5%
Anti-dyslipidaemic agents	3281	43.1%	3582	41.6%

Table S6. Adherence and discontinuation during follow-up for patients with proxy od AMD.

	**Overall**		**Aflibercept**		**Bevacizumab**		**Dexamethasone**		**Ranibizumab**		**Pegaptanib**	
**TOTAL**	8025		2363		295		808		4453		106	
**Number of injections in the follow-up period, Mean (SD)**												
Year 1 (including the index date injection)	3.9 (2.1)		4.6 (2.1)		3.6 (2.1)		1.9 (1.2)		3.9 (2.0)		3.8 (1.7)	
Year 2	1.4 (2.0)		1.7 (2.2)		1.1 (1.9)		0.7 (1.3)		1.3 (2.0)		1.1 (1.8)	
Year 3 (cohort with index date<=31/12/18 only)	1.1 (1.8)		1.4 (2.1)		0.8 (1.5)		0.5 (1.1)		1.0 (1.8)		0.6 (1.3)	
**Number of contact in the follow-up period, Mean (SD) (specialist examinations)**												
Year 1 (including the index date injection)	9.0 (6.7)		9.0 (6.3)		6.2 (4.3)		6.1 (4.4)		9.6 (7.1)		14.0 (8.7)	
Year 2	3.7 (5.1)		3.7 (4.7)		2.6 (3.9)		2.7 (3.7)		3.9 (5.4)		6.0 (7.4)	
Year 3 (cohort with index date<=31/12/18 only)	2.8 (4.5)		3.0 (4.5)		1.8 (2.9)		1.8 (3.0)		3.0 (4.7)		3.5 (5.0)	
**Number of subjects with (at least) the first 3 doses in 90 days, N (%)**	3601	44,9%	1272	53,8%	80	27,1%	4	0,5%	2223	49,9%	22	20,8%
**At least 60 days without contact in the first year of follow-up and at least 90 days in the second, N (%)**	5492	68,4%	1876	79,4%	186	63,1%	429	53,1%	2928	65,8%	73	68,9%
**At least 90 days without contact in the first year of follow-up and at least 180 days in the second, N (%)**	4205	52,4%	1283	54,3%	129	43,7%	427	52,8%	2315	52,0%	51	48,1%

Table S7. History of comorbidities for patients with proxy od AMD.

	**Overall**		**Aflibercept**		**Bevacizumab**		**Dexamethasone**		**Ranibizumab**		**Pegaptanib**	
**TOTAL**	8025		2363		295		808		4453		106	
**Charlson Comorbidity Index**												
mean (SD)	0.2 (0.8)		0.2 (0.9)		0.2 (0.8)		0.3 (0.9)		0.2 (0.8)		0.3 (0.8)	
**Number of comorbidities (one of those present in Table)**												
0	5567	69,4%	1641	69,4%	218	73,9%	542	67,1%	3092	69,4%	74	69,8%
1-2	2224	27,7%	644	27,3%	73	24,7%	237	29,3%	1242	27,9%	28	26,4%
3-5	231	2,9%	76	3,2%	4	1,4%	29	3,6%	118	2,6%	4	3,8%
6+	3	0,0%	2	0,1%	0	0,0%	0	0,0%	1	0,0%	0	0,0%
**Psychiatric or mental health conditions, N (%)**												
Anxiety	63	0,8%	13	0,6%	0	0,0%	9	1,1%	41	0,9%	0	0,0%
Bipolar disease	12	0,1%	3	0,1%	0	0,0%	2	0,2%	7	0,2%	0	0,0%
Delirium	3	0,0%	1	0,0%	0	0,0%	1	0,1%	1	0,0%	0	0,0%
Dementia	12	0,1%	1	0,0%	0	0,0%	3	0,4%	7	0,2%	1	0,9%
Depression	18	0,2%	5	0,2%	0	0,0%	3	0,4%	10	0,2%	0	0,0%
Non-schizophrenic psychosis	7	0,1%	2	0,1%	0	0,0%	1	0,1%	4	0,1%	0	0,0%
Schizophrenia	4	0,0%	0	0,0%	0	0,0%	1	0,1%	3	0,1%	0	0,0%
**Cardio/cerebrovascular diseases, N(%)**												
Arrhythmia	115	1,4%	47	2,0%	0	0,0%	15	1,9%	52	1,2%	1	0,9%
Congestive heart failure	98	1,2%	24	1,0%	3	1,0%	9	1,1%	60	1,3%	2	1,9%
Ischemic heart disease	370	4,6%	118	5,0%	12	4,1%	32	4,0%	195	4,4%	13	12,3%
Stroke	93	1,2%	31	1,3%	5	1,7%	12	1,5%	44	1,0%	1	0,9%
Hypertension	2037	25,4%	587	24,8%	67	22,7%	223	27,6%	1133	25,4%	27	25,5%
Peripheral arterial disease	6	0,1%	3	0,1%	0	0,0%	1	0,1%	2	0,0%	0	0,0%
Valve disorders	107	1,3%	30	1,3%	1	0,3%	11	1,4%	63	1,4%	2	1,9%
Venous thrombosis embolism	17	0,2%	4	0,2%	1	0,3%	4	0,5%	8	0,2%	0	0,0%
**Other conditions, N(%)**												
Diabetes	0	0,0%	0	0,0%	0	0,0%	0	0,0%	0	0,0%	0	0,0%
COPD	168	2,1%	53	2,2%	4	1,4%	15	1,9%	93	2,1%	3	2,8%
Osteoporosis	58	0,7%	23	1,0%	2	0,7%	2	0,2%	30	0,7%	1	0,9%
Parkinson’s disease	19	0,2%	5	0,2%	0	0,0%	2	0,2%	11	0,2%	1	0,9%
Epilepsy	34	0,4%	14	0,6%	0	0,0%	6	0,7%	13	0,3%	1	0,9%
Hip fracture	19	0,2%	8	0,3%	1	0,3%	2	0,2%	8	0,2%	0	0,0%
Pneumonia	112	1,4%	32	1,4%	3	1,0%	11	1,4%	66	1,5%	0	0,0%

Table S8. History of concomitant drugs for patients with proxy od AMD.

	**Overall**		**Aflibercept**		**Bevacizumab**		**Dexamethasone**		**Ranibizumab**		**Pegaptanib**	
**TOTAL**	8025		2363		295		808		4453		106	
**Concomitant drugs**												
Mean ± SD	7.5 (4.5)		7.4 (4.3)		7.6 (4.4)		7.8 (4.2)		7.6 (4.7)		8.0 (4.7)	
Median (IQR)	7.0 (4.0-10.0)		7.0 (4.0-10.0)		7.0 (5.0-10.0)		7.0 (5.0-10.0)		7.0 (4.0-10.0)		7.0 (5.0-10.0)	
Min - Max	0.0-52.0		0.0-28.0		0.0-36.0		0.0-25.0		0.0-52.0		0.0-25.0	
**Number of different drugs, N (%)**												
0	221	2,8%	68	2,9%	5	1,7%	13	1,6%	130	2,9%	5	4,7%
1-2	748	9,3%	224	9,5%	32	10,8%	56	6,9%	432	9,7%	4	3,8%
3-5	1925	24,0%	563	23,8%	60	20,3%	203	25,1%	1077	24,2%	22	20,8%
6-9	2771	34,5%	843	35,7%	116	39,3%	289	35,8%	1483	33,3%	40	37,7%
10+	2360	29,4%	665	28,1%	82	27,8%	247	30,6%	1331	29,9%	35	33,0%
**Type of drug, N (%)**												
Digoxin	120	1,5%	28	1,2%	7	2,4%	4	0,5%	79	1,8%	2	1,9%
NSAIDs	3563	44,4%	988	41,8%	141	47,8%	347	42,9%	2037	45,7%	50	47,2%
Low dosage aspirin	2484	31,0%	718	30,4%	93	31,5%	306	37,9%	1328	29,8%	39	36,8%
Antibacterial	4932	61,5%	1388	58,7%	196	66,4%	512	63,4%	2766	62,1%	70	66,0%
Anti-thrombotic	3483	43,4%	1017	43,0%	127	43,1%	436	54,0%	1849	41,5%	54	50,9%
Drugs for peptic ulcers	4506	56,1%	1297	54,9%	168	56,9%	460	56,9%	2520	56,6%	61	57,5%
Organic nitrates	328	4,1%	81	3,4%	16	5,4%	24	3,0%	194	4,4%	13	12,3%
Corticosteroids	1480	18,4%	441	18,7%	46	15,6%	181	22,4%	794	17,8%	18	17,0%
Antihypertensives	424	5,3%	118	5,0%	14	4,7%	52	6,4%	234	5,3%	6	5,7%
Anti-dyslipidaemic agents	2845	35,5%	848	35,9%	102	34,6%	282	34,9%	1562	35,1%	51	48,1%

Table S9. History of proxy of diabetes-related eye disease, glaucoma, use of ophthalmic services and binocularity by age

	**64-**		**65-69**		**70-74**		**75-79**		**80-84**		**85+**	
**TOTAL**	3504		2026		2818		3174		2764		1940	
**Proxy of diabetes-related eye disease in the look-back period, No. of subjects, N (%)**												
Diabetes	1431	40.8%	919	45.4%	1066	37.8%	967	30.5%	756	27.4%	406	20.9%
Argon-laser retina (and Laser photocoagulation)	492	14.0%	221	10.9%	217	7.7%	162	5.1%	80	2.9%	33	1.7%
Younger than 55 at first injection	1310	37.4%	0	0.0%	0	0.0%	0	0.0%	0	0.0%	0	0.0%
Any proxy among the previous	2363	67.4%	958	47.3%	1119	39.7%	1029	32.4%	788	28.5%	422	21.8%
**Glaucoma, N (%)**	123	3.5%	96	4.7%	98	3.5%	120	3.8%	108	3.9%	72	3.7%
**Use of ophthalmic services during in the look-back period, Mean (SD)**												
Specialist encounter	1.9 (2.1)		2.1 (2.0)		2.1 (2.1)		2.1 (2.1)		2.0 (2.0)		1.8 (1.9)	
OCT	0.0 (0.0)		0.0 (0.0)		0.0 (0.0)		0.0 (0.0)		0.0 (0.0)		0.0 (0.0)	
Fluorescence imaging	0.7 (0.8)		0.7 (0.9)		0.7 (0.9)		0.6 (0.9)		0.6 (0.9)		0.5 (0.8)	
Fluorescence imaging with indocyanine	0.0 (0.0)		0.0 (0.0)		0.0 (0.0)		0.0 (0.0)		0.0 (0.0)		0.0 (0.0)	
Fundus photography	0.0 (0.2)		0.0 (0.2)		0.0 (0.1)		0.0 (0.1)		0.0 (0.1)		0.0 (0.1)	
**Possible binocularity during the first year of follow-up period, No of subjects (%)**	393	11.2%	208	10.3%	286	10.1%	314	9.9%	253	9.2%	158	8.1%

Table S10. History of proxy of diabetes-related eye disease, glaucoma, use of ophthalmic services and binocularity by gender

	**Male**		**Female**	
**TOTAL**	7621		8605	
**Proxy of diabetes-related eye disease in the look-back period, No. of subjects, N (%)**				
Diabetes	3094	40.6%	2451	28.5%
Argon-laser retina (and Laser photocoagulation)	728	9.6%	477	5.5%
Younger than 55 at first injection	676	8.9%	634	7.4%
Any proxy among the previous	3649	47.9%	3030	35.2%
**Glaucoma, N (%)**	287	3.8%	330	3.8%
**Use of ophthalmic services during in the look-back period, Mean (SD)**				
Specialist encounter	2.0 (2.0)		2.0 (2.1)	
OCT	0.0 (0.0)		0.0 (0.0)	
Fluorescence imaging	0.7 (0.9)		0.6 (0.9)	
Fluorescence imaging with indocyanine	0.0 (0.0)		0.0 (0.0)	
Fundus photography	0.0 (0.2)		0.0 (0.2)	
**Possible binocularity during the first year of follow-up period, No of subjects (%)**	752	9.9%	860	10.0%
